# Evaluation of a medication monitor-based treatment strategy for drug-sensitive tuberculosis patients in China: study protocol for a cluster randomised controlled trial

**DOI:** 10.1186/s13063-018-2650-3

**Published:** 2018-07-25

**Authors:** James J. Lewis, Xiaoqiu Liu, Zhiying Zhang, Bruce V. Thomas, Anna Vassall, Sedona Sweeney, Xu Caihong, Hu Dongmei, Li Xue, Gao Yongxin, Shitong Huan, Jiang Shiwen, Katherine L. Fielding

**Affiliations:** 1 MRC Tropical Epidemiology Group, Department of Infectious Disease Epidemiology, London School of Hygiene & Tropical Medicine, London, UK; 2 TB Centre, London School of Hygiene and Tropical Medicine, London, UK; 3 National Center for Tuberculosis Control and Prevention, Chinese Center for Disease Control and Prevention, Beijing, China; 4 PATH, Beijing, China; 5 The Arcady Group, Richmond, VA USA; 6 Department of Global Health and Development, London School of Hygiene and Tropical Medicine, London, UK; 7 Bill & Melinda Gates Foundation, China Office, Beijing, China

**Keywords:** Tuberculosis, medication monitor, eHealth, China, trial, cluster randomised, pragmatic

## Abstract

**Background:**

Treatment for drug-sensitive tuberculosis (TB) is taken for at least 6 months and problems with adherence are common. Therefore, there is substantial interest in the possible use of eHealth interventions to support patients to take their treatment. Electronic medication monitors have been shown to improve adherence to TB medication, but the impact on clinical outcomes is unknown. We aim to evaluate the impact of a medication monitor-based treatment strategy for drug-sensitive TB patients on a composite poor outcome measured over 18 months from start of TB treatment.

**Methods/design:**

We will conduct an open, pragmatic, cluster randomised superiority trial, with 24 counties/districts in three provinces in China, randomised 1:1 to implement the intervention or standard of care. Adults (aged ≥ 18 years) with a new episode of GeneXpert-positive and rifampicin-sensitive pulmonary TB, who plan to be in the study area for the next 18 months, and will receive daily fixed-dose combination tablets for 6 months of treatment are eligible. The intervention is centred around a medication monitor that holds a 1-month supply of medication and has three key functions: as an audio and visual reminder for patients to take their daily medication; reminds patients of upcoming monthly visit; and records date and time whenever the box is opened. At the monthly follow-up visit, the doctor downloads these data to generate a graphical display of the last month’s adherence record for discussion with the patient and potentially to switch the patient to more intensive management. The primary outcome is a composite poor outcome measured over 18 months from start of TB treatment, defined as either of poor outcome at the end of treatment (death, treatment failure, or loss to follow-up) or subsequent recurrence (culture positive for TB at 12 or 18 months or re-starting TB treatment in the follow-up period). An economic evaluation will also be conducted as part of this study.

**Discussion:**

This trial will assess whether a medication monitor-based treatment strategy can improve clinical outcomes for TB patients. Several trials of other eHealth interventions for TB treatment are ongoing and are summarised in this paper. This trial will provide an important part of the emerging evidence base for the potential of eHealth to improve TB treatment outcomes.

**Trial registration:**

This trial was registered with Current Controlled Trials (identifier: ISRCTN35812455). Registered on May 19, 2016.

**Electronic supplementary material:**

The online version of this article (10.1186/s13063-018-2650-3) contains supplementary material, which is available to authorized users.

## Background

### Background and rationale

Tuberculosis (TB) remains one of the world’s deadliest communicable diseases. In 2015, an estimated 10.4 million people developed TB and 1.4 million died from the disease [1]. China accounts for the third highest number of cases worldwide, with 0.92 million estimated incident cases in 2015 [1]. The World Health Organization (WHO) declared a global TB control emergency in April 1993, with a global Directly Observed Treatment Short-course strategy being proposed as a solution. A key point of this strategy is directly observed treatment (DOT), designed specifically to strengthen treatment adherence. The WHO defines DOT as 6–8 months’ worth of regular treatment for TB patients who have already been found to be infectious, along with direct observation of patient drug intake during the intensive phase, at the very least. Implementation of the Directly Observed Treatment Short-course strategy commenced in China in 1992 and covered the entire country by 2005 [2]. However, a meta-analysis of studies from China reported that 52% of the TB patients were on self-administered treatment, 27% were observed by family members and only 20% were observed by health workers [3]. In the 2010 National TB Prevalence Survey in China, 20% of TB patients treated by the public health system – using national TB case-management approaches – were lost to follow-up or were not taking their medications regularly [4]. As for many low- and middle-income countries, it is difficult to fully implement DOT management for the treatment of TB in China, complicated by the high cost of healthcare and differences in economic levels, geography, availability of transportation, and patient preferences [3].

In response to the problems of implementing DOT, current developments in treatment management involve the utilisation of eHealth technology and research to discover more effective and efficient ways of ensuring that patients take their medication, as highlighted by the WHO Global Task Force on Digital Health for TB [5]. The 2017 update to the WHO’s TB treatment guidelines suggests that tracers (such as mobile phone short message service (SMS)) and/or digital medication monitors may be offered to patients on TB treatment and that video-observed treatment (VOT) can replace DOT under some circumstances [6]. However, both are conditional recommendations with “*very low certainty in the evidence*”. A systematic review of SMS use for promoting adherence to TB medication found four studies with a control group, of which only one was a randomised controlled trial and this trial had only 37 patients [3, 7].

In our previous pragmatic, cluster-randomised trial, 36 districts/counties within four provinces of China were randomised to one of four case-management approaches utilising SMS, a medication monitor, both, or neither (control) [8]. Treatment adherence was the primary endpoint. The intervention arms had reminders for drug intake, reminders for monthly follow-up visits, and a recommendation to switch patients with adherence problems to more intensive management or DOT. The percentage of patient-months on TB treatment where at least 20% of doses were missed was 29.9% in the control arm, 27.3% in the SMS arm (adjusted mean ratio (aMR) of 0.94, 95% confidence interval (CI) 0.71–1.24), 17.0% in the medication monitor arm (aMR 0.58, 95% CI 0.42–0.79), and 13.9% in the combined arm (aMR 0.49, 95% CI 0.27–0.88). Patient loss to follow-up was lower in all three intervention arms, but there was only statistical evidence for this reduction in the SMS arm (aMR 0.42, 95% CI 0.18–0.98). Equipment malfunction or operation error was reported in all study arms. Based on these results, the use of a medication monitor shows great promise. A new, improved version of the medication monitor has been developed [9], including generation of a graphical display of a patient’s detailed dosing history at monthly follow-up visits to facilitate discussion between the patient and doctor. However, it remains to be seen whether these improvements in adherence, and possibly in retention, translate into meaningful improvements in clinical outcomes.

The overall aim of the trial is to investigate whether drug-sensitive pulmonary adult TB patients whose treatment strategy includes a medication monitor for daily drug dosing reminders and monitoring of adherence patterns, together with targeted intensive management of patients with poor adherence patterns (intervention arm), have better clinical and adherence outcomes compared with patients managed according to standard of care (control arm).

### Rationale for a randomised controlled trial

Although the first trial showed promising results, our primary outcome was adherence to treatment and the relationship between adherence and clinical outcomes is likely to be non-linear. We had included a secondary outcome of poor treatment outcome, though the study was underpowered for this and the outcome was based on routine data collected at the end of treatment, which is considered a poor proxy for true cure of TB. The intervention has now changed to include an explicit discussion of detailed dosing history between patients and doctors as well as an improved medication monitor. Although the improved medication monitor remains at low cost per patient, rolling this out for almost one million TB patients annually would still require a substantial financial investment and increased training. Hence, strong evidence is needed that this would lead to a substantial improvement in patient clinical outcomes, which can best be obtained from a randomised controlled trial.

### Rationale for a cluster randomised trial

There is debate as to whether trials of health service delivery should be individually or cluster randomised [10, 11]. Cluster randomised trials require larger sample sizes than comparable individually randomised trials due to the lack of independence, and resulting intra-cluster correlation, between observations within clusters. As they typically randomise a much smaller number of units, cluster randomised trials also have an increased chance of baseline imbalance by study arm. However, if the intervention is to be delivered at the level of a cluster (for example, a clinic or a district), then the trial needs to be cluster randomised.

In this trial, all patient recruitment, follow-up and data collection will be performed by routine staff and it is thought to be substantially more complicated to request that they also undertake randomisation of patients into two trial arms. The intervention requires changes to delivery of care, which will be easier to implement if intervention and control patients are not both managed in the same clinic. Under individual randomisation, patients treated in the same clinic might discuss with other patients regarding the adherence technology and the subsequent advice received following discussion of detailed dosing history, both of which would cause a dilution of the effect size. In addition, staff might also change their behaviour and become more likely to monitor and discuss adherence with patients randomised to the control arm, thereby changing the usual standard of care, also diluting the effect size. For these reasons, we decided to randomise at the level of counties/districts as this is the smallest level at which comprehensive TB care is delivered (within China the distinction between counties and districts as administrative areas is defined as less than, or more than, half the population residing in an urban area).

### Hypothesis

We hypothesise that managing drug-sensitive pulmonary adult TB patients using a treatment strategy that includes a medication monitor with daily reminders of drug dosing, monthly reminders for pharmacy refills, and the monitoring of adherence patterns, as well as targeted intensive management of patients with poor adherence patterns, will result in better clinical and adherence outcomes.

## Methods/design

### Study design

This is a two-arm, unblinded, pragmatic, cluster randomised superiority trial, with equal numbers of clusters randomised to intervention and control arms. The unit of randomisation will be counties/districts in three provinces in China with the distinction between counties and districts being one of degree of urbanisation.

### Study setting

National TB Control Programme guidelines in China require drug-sensitive TB patients to take fixed-dose combination tablets daily for 6 months. Each county/district has one designated hospital or TB dispensary in which patients initiate TB treatment and then have monthly consultations with a TB doctor to discuss side effects and adherence, and to obtain the next month’s supply of tablets.

### Intervention arm

**Fig. 1:**
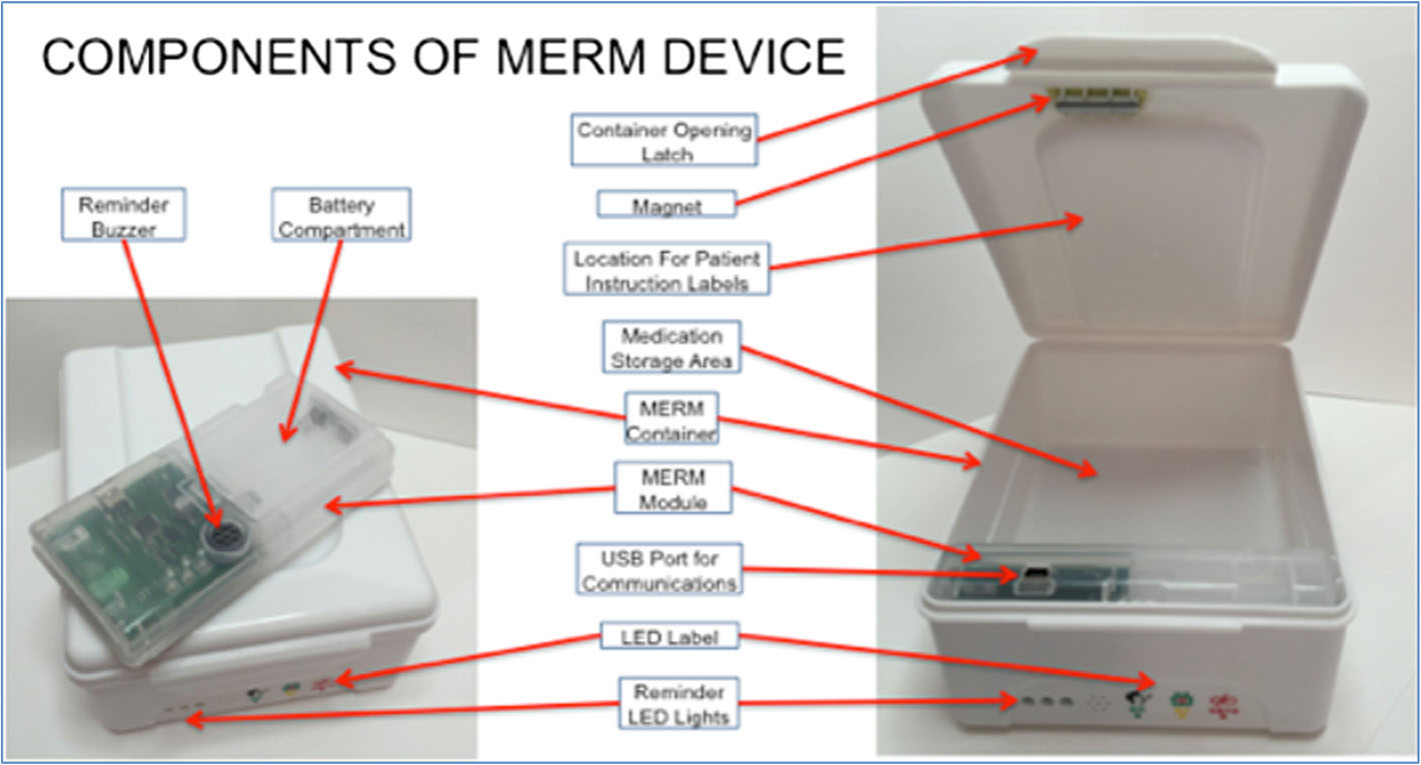
Graphical illustration of the medication event reminder monitor (MERM) to be used in this trial (dimensions are: height 71.4 mm x width 129 mm x length 166.9 mm)

Patients will be asked to keep their monthly supply of medication in the medication event reminder monitor (MERM), which has three key functions: an audio and visual reminder for patients to take their daily medication; reminds patients of upcoming monthly visits; and to record date and time whenever the box is opened (Table 1; Fig. 1) [9]. The MERM used in this study is the evriMED 500 (Wisepill, Cape Town, South Africa). At the monthly follow-up visit, their managing doctor downloads these data to a computer system that generates a graphical display of the last month’s adherence record, which doctors use to discuss adherence issues with the patient and to decide whether to switch the patient to more intensive management.

**Table 1:** Description of intervention versus standard of care (control) arms

Service contents	Intervention arm	Control arm
Supervision method for daily dosing	Patient receives daily reminders for drug intake by the MERM through a buzzer sound and green light, which are active for 5 min then silent for 5 mins, and then is repeated twice. If the MERM is opened during this period, then the alarm is cancelled until the next day. The time of the reminder is set by the doctor at enrolment and can be changed at subsequent follow-up visits.	Patients choose one of the three methods of adherence supervision in consultation with their doctor at the start of treatment: direct observation by (1) healthcare worker or (2) family member; or self-administered.
Follow-up visit reminder	A yellow light on the MERM is used to remind patients to attend their monthly follow-up visit. The light comes on daily, at an agreed time, for 30 mins, for 3 days before their scheduled visit date. A pictogram label on the MERM indicates that sputum should be collected at clinic visits at 2, 5, and 6 months.	No reminder. Pictogram labels are not used on the MERM for patients in the control arm.
Monthly follow-up patient visit to the doctor	The TB doctor at the county (district) level exports data on date/time of the box being opened from the MERM and a graphical display of the dosing history for the last month is generated. The doctor shows the graphical display to the patient, and discusses the patient’s drug-intake summary and the importance of timely drug-intake. A printed copy of graphical display summary is given to the patient, where feasible. Based on the MERM data from the last month, the doctor determines whether an adjustment to the way of managing patient medication is required. Actions are described below.	Patients are seen at the monthly follow-up visits by the TB doctor at the county (district) level. If the TB treatment record card indicates doses have been missed, the doctor asks the patient about why drugs have been missed and discusses the importance of timely and regular drug intake. The doctor does not have access to the MERM data.
Judgment and handling of missing doses	The doctor assesses adherence using data from the MERM, excluding time periods when the patient had been in hospital or travelling. If: < 20% doses missed: reasons for doses missed are ascertained and the patient educated about keeping healthy. No change to the management of the patient. 20–50% doses missed (first occasion): Township doctors are asked to visit the patient every 2 weeks and village doctors are asked to visit the patient every week to support medication adherence. > 50% doses missed or 20–50% doses missed (second occasion): management mode is changed to “taking medicine in the presence of medical staff”, namely, village doctors are required to directly supervise patients to take medicine daily.	No specific requirement.
Doctor to patient visit	(1) The CDC doctor visits each patient once during the intensive and continuous phases. At the visit, the patient's adherence and the use of MERM is discussed. If the patient is reluctant to take treatment, the reason is identified and information is given to the patient on keeping healthy. (2) Doctors from community service centres visit the patient once a month to confirm use of the MERM and monitor adverse reactions. Any errors in using the MERM or any serious adverse reactions are immediately reported to the organisation for TB prevention at the county (district) level.	Standard NTP practice: (1) The CDC doctor visits each patient once during the intensive and continuous phases. The doctor asks about the patient’s drug adherence, gives advice about timely and regular drug intake, and educates the patient about keeping healthy. (2) Doctors from community service centres visit patients who are self-supervised/family member supervision every 10 days in the intensive phase, and once a month in the continuous phase.

*CDC* Center for Disease Control, *MERM* medication event reminder monitor, *NTP* National Tuberculosis Programme

In both arms the following occurs:

If a patient does not attend the scheduled follow-up visit, the hospital/dispensary doctor, nurse or other staff member contacts the patient (using patient or family member phone) and asks them to return to the hospital/dispensary for follow-up. If a patient does not attend a follow-up visit 3 days after the scheduled date, the hospital/dispensary doctor informs the CDC, who in turn informs the village doctor. The village doctor is required to visit the patient and supervise them to visit the designated medical institution to get medications or receive sputum examination within 24 h after receiving the follow-up notification

### Standard of care

Patients, in consultation with the doctor, choose whether to take their tablets under direct observation by a healthcare worker, direct observation by a family member, or through self-administration (Table 1). After each dose is taken, a mark is put on a TB treatment record card, which is shown to the doctor at the monthly follow-up visit as a way of determining adherence.

### Selection of study counties/districts

Counties/districts were selected from four prefectures (Hangzhou, Weizhou, Jilin and Ganzhou) in three provinces (Zhejiang, Jilin and Jiangxi) chosen to reflect a variety of settings. Within these four prefectures, 24 counties/districts were chosen with access to GeneXpert MTB/RIF and culture facilities, at least 300 pulmonary TB patients treated in 2014, implementing daily TB treatment in a TB-designated hospital or TB dispensary, and that were willing to participate in this study and were not already participating in another study.

### Study population

The study population comprises a consecutive sample of adults (≥ 18 years) with a new episode of Gene Xpert-positive and rifampicin-sensitive pulmonary TB. In order to maximise generalisability, we plan to make the trial as inclusive as possible. However, the study procedures require that the patient is likely to be in the study area for the 18 months following inclusion in the study, receives daily fixed-dose combination tablets, is not known at the start of treatment to need more than 6 months of treatment or to be hospitalised for more than 2 months, and is not known to be HIV positive.

### Trial outcomes

The primary outcome is a composite poor outcome measured over 18 months from start of TB treatment, defined as either of poor outcome at the end of treatment (death, treatment failure or loss to follow-up) or subsequent recurrence (culture positive for TB at 12 or 18 months or re-starting TB treatment in the follow-up period). Secondary outcomes are three clinical outcomes and two adherence outcomes: poor outcome at the end of treatment; composite poor outcome measured at 12 months; lost to follow-up during treatment; percentage of months in which the patient missed at least 20% of doses, measured using data from the MERM; and percentage of total doses missed. Cost-effectiveness outcomes include mean cost per patient treatment month for each arm, and incremental cost of the intervention per patient completing treatment, per death and per disability-adjusted life year averted.

### Economic evaluation

An economic evaluation will be conducted from a societal perspective, estimating the incremental cost per disability-adjusted life year averted of MERM compared to the standard of care. Data will be collected on two types of incremental costs, namely (1) incremental costs of MERM implementation and (2) incremental cost of TB treatment in both MERM and the standard of care. Analysis will be carried out in two stages. First, a within-trial analysis will be conducted comparing cost-effectiveness within the trial population and period, using statistical methods to estimate the probability that the intervention is cost-effective. The second stage will use a decision analytical model to extend these results over time to include downstream costs (such as the further treatment of recurrences).

### Sample size considerations

Recently completed phase III TB treatment trials, conducted under clinical trial conditions, found percentages with poor outcome at end of treatment of 8.5% to 11.2%, and by 18 months from enrolment ranging from 13.2% to 15.7%, in the control arm [12–14]. As these data were collected under clinical drug trial conditions we would expect poor outcomes to be higher in a pragmatic setting. Data from our first adherence trial in China found a combined poor outcome at the end of treatment of 9.2%. This was in the absence of culture to define treatment success and it is therefore reasonable to expect a higher risk of poor outcome when using culture to define end of treatment outcomes. Assuming 12 clusters per arm, 125 patients per cluster, 5% of individuals whose outcome at 18 months cannot be ascertained, a composite poor outcome estimate of 18% in the control arm, and taking account of the clustered design using a coefficient of variation of 0.25, there will be 92% power to assess a 40% reduction in poor outcome. In our first adherence trial comparing the medication monitor arm to the standard of care arm, poor adherence was reduced by 42% (95% CI 21–58% reduction) and poor treatment outcome by 29% (95% CI 51% increase to 67% reduction); therefore, a reduction of 40% should be achievable with an improved intervention.

### Randomisation

Unstratified, restricted randomisation was used to improve baseline balance, as is common for cluster randomised trials with a small number of clusters. The restrictions were: within each of the four prefectures, the difference in number of intervention and control clusters should be at most one; there should be seven clusters with a designated hospital and five with a TB dispensary in each arm; the difference in the number of intervention and control arm clusters should be at most one in each of the seven urban and 17 rural clusters; and the difference in the average number of smear-positive TB cases notified in each cluster in 2015 between intervention and control arm clusters should be at most 10 cases. Applying these criteria to 10,000 randomly generated allocations left 102 acceptable allocations (1.02%), giving a restriction factor of 0.990 (95% CI 0.988–0.992). Of 2,704,156 possible allocations, this left approximately 27,582 acceptable allocations (95% CI 22,510–33,446). The proportion of 5000 randomly generated, acceptable allocations for which a pair of clusters were in the same arm varied between 0.261 and 0.770, suggesting that the most restrictive criteria would still allow for appropriate statistical inference. One of these 5000 acceptable allocations was then chosen at random in Stata version 14 by the statistician (author JJL) and communicated in this unblinded trial.

### Study procedures

**Fig. 2:**
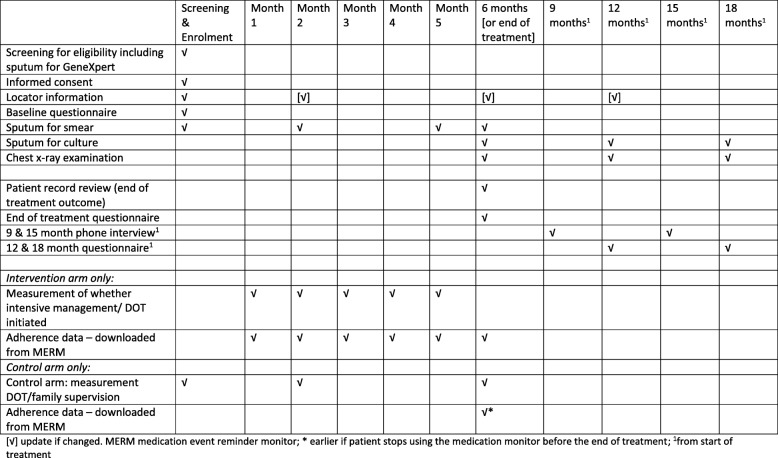
SPIRIT figure – summary of study procedures for intervention and control arms

Consecutive adult patients starting TB treatment at the TB dispensary/hospital in the county/district will be screened by their treating doctor (as trained by the study team) and, if eligible, offered enrolment in the study. Following informed consent (see Additional file 1 for informed consent form and patient information sheet), participants will complete a questionnaire and be asked to give a sputum specimen for smear microscopy and GeneXpert (see Fig. 2 for a summary of study procedures for intervention and control arms), and Additional file 2 for completed SPIRIT checklist).

Participants in both the intervention and control arms will be given a MERM box and 30 days of medication, which they will be asked to keep in the MERM. The box records the date and time of each opening. Participants will return to the TB dispensary/hospital for each of six monthly follow-up visits (months 1–6). Patients in the intervention arm will be required to bring their MERM to each monthly follow-up visit, at which the doctor or designee will connect it to the computer and download the data. In the control arm, the MERM is in silent mode (no audio or visual reminders) and data from the box will be downloaded once, at the end of treatment or earlier if the patient stops using the MERM before the end of treatment; the doctor will not be able to access these adherence data. In either arm, if applicable, the doctor or designee will assist the patient to fix any problems with the box and, if necessary, to replace it.

Sputum will be collected at the end of the second month for an exploratory endpoint of 2-month smear conversion and again at the end of treatment for culture (using Lowenstein–Jensen) and smear to enhance the definitions of treatment ‘cure’ and ‘failure’.

Following the end of treatment, the treatment outcome, as recorded by the National TB Control programme, will be abstracted. Patients will be seen at the TB hospital/dispensary at two further follow-up visits, at 12 and 18 months after start of treatment, for a chest x-ray and to collect a sputum specimen for culture in order to measure recurrence. Patients will also be asked about TB symptoms and whether they have re-started TB treatment. The hospital/dispensary doctor, nurse or other staff member will help facilitate patient attendance at these post-treatment follow-up visits. In addition, these patients will be telephoned at months 9 and 15 after the start of treatment and asked about TB symptoms and whether they have re-started TB treatment. Patients with TB symptoms will be advised to attend a healthcare clinic. If a patient has restarted TB treatment, information from the TB register and TB diagnosis will be abstracted.

Participants are free to withdraw from the study at any time and those in the intervention arm can stop using the MERM, whilst remain in the study, at any time. Participants will also be withdrawn from the study if they plan to move away or travel continuously for more than 1 month, or if they are no longer using daily fixed-dose combination tablets. All participants who withdraw early will be asked if we can still collect their treatment outcomes and contact them for post-treatment follow-up. No other changes to care are prohibited during the trial.

A pilot study enrolled 55 patients in four counties/districts before commencing enrolment into the trial to field test the MERM and associated software, the recruitment process, the monthly adherence feedback given to patients by the managing Doctor and case report forms. Following the pilot study, all 24 counties/districts had a 3-month run-in period to help staff familiarise themselves with the recruitment and follow-up processes and ensure all monitoring processes are being implemented.

### Data management

The treating doctors will be trained and responsible for collecting written, informed consent, assessing eligibility, and capturing all data into a bespoke database. A password will be required to gain access to the database. Data will be validated on entry, using range and consistency checks. Logical data checks will also be performed on the data. Incomplete and incorrect data queries will be sent back to sites electronically for error resolution. All study records will be managed in a secure and confidential fashion. All records will be stored at the participating TB clinics and offices at provincial level in locked filing cabinets and access to the records will be restricted to specified study team members. Case report forms will be identified using the participant’s study number only, with locator information stored separately. When datasets are generated for data analysis, personal identifiers will be removed. All records will be archived in a secure storage facility for at least 10 years after the completion of the study.

### Statistical analysis

Analyses will use an intention-to-treat approach with methods appropriate for the clustered randomised trial design, giving each cluster equal weight [10]. Quantitative outcomes will be summarised as the mean for each cluster and the difference of means for intervention versus standard of care arm. For binary outcomes or rate outcomes, the overall risk (rate) for each cluster will be calculated as well as the ratio for intervention versus standard of care arm. An adjusted analysis will be conducted to control for baseline imbalance of patient or district/county level factors across control and intervention arms. Subgroup analyses will be conducted for the primary outcome and may include urban/rural, gender, literacy levels, migrant/non-migrant and type of healthcare provider. A statistical analysis plan will be finalised prior to the end of data collection.

### Ethics and dissemination

The trial has approval from the Institutional Review Board of the Chinese Center for Disease Control and Prevention (ref: 201603) and the London School of Hygiene & Tropical Medicine Ethics Committee (ref: 10665). It is registered with Current Controlled Trials (identifier ISRCTN35812455). Participants in both intervention and control arms will receive around 200 RMB (in total; approximately US$30) if they attend for study review visits at 12 and 18 months after enrolment, as compensation for their time and travel expenses. In addition, all patients will benefit from a free GeneXpert test at enrolment, allowing quick diagnosis of drug resistance to rifampicin, as well as free cultures at the end of treatment and at 12 and 18 months. Written, informed consent will be sought from potential participants by their treating doctor (as trained by the study team), using standard consent forms and information sheets available in Chinese Mandarin.

The trial results will be communicated to stakeholders through dissemination meetings. Investigators will present results at relevant conferences and submit manuscript(s) to peer-reviewed journals. Public access to the participant-level dataset of main trial results and statistical code will be made available.

### Trial governance

Xiaoqiu Liu is the Chief Investigator and is employed by the Chinese Center for Disease Control and Prevention, which is the trial sponsor. The trial sponsor had no role in the design of this study and will not have any role during its execution, analyses, interpretation of the data, or decision to submit results.

The trial investigator team is responsible for the trial design, leading the implementation, data analysis, publication, and determining authorship eligibility guidelines, while a trial implementation team is responsible for site training, study monitoring, auditing trial conduct in site visits once every 3 months, and day-to-day implementation of the trial. A trial steering committee oversees the trial, monitors its progress and provides advice to the Chief Investigator and trial investigator team.

The trial does not have a Data Monitoring Committee, as any potential harms to patients are thought to be low risk. The Patient Information Sheet contains two telephone numbers for the Principal investigator (author XL), in case the patient wants to ask anything, and this is the primary means by which patients can report problems with trial conduct. The Trial Steering Committee will review progress data and will thus partially function as a Data Monitoring Committee. Recruitment will take approximately 2 years, and for each individual in the study there will be a 6-month treatment period followed by a further 12 months of follow-up. At the time that recruitment ends, there will only be primary outcome data for approximately a quarter of participants. Hence, there is no planned interim analysis. Any major changes to the study would be updated in the protocol and trial registration, reported to the ethics committees for approval, and communicated to the trial steering committee at the next meeting.

## Preliminary data from the run-in period

A 3-month run-in period was conducted in all 24 clusters. TB patients recruited during this period will be followed up at the end of treatment but not contribute to the trial outcomes. Overall, 485 patients have been recruited, of which 73% are male, median age 40 years (interquartile range: 27–57 years), 7% are illiterate and 64% had smear-positive TB (Table 2). Most characteristics were similar to those observed in the first study, but patients in this study were less likely to be living in their place of registration (69% vs. 91%) and were more likely to have smear-positive TB (64% vs. 36%).

**Table 2:** Data from run-in period from the 24 clusters (*n* = 485)

Characteristic	Category	Number	Percent
Gender	Female	132	27.2%
Age, years	18–29	152	31.3%
	30–39	87	17.9%
	40–59	149	30.7%
	60+	97	20.0%
Farmer	Yes	226	46.6%
Education, highest level	Illiterate	36	7.4%
	Primary School	134	27.6%
	Junior Middle School	178	36.7%
	High School (Technical School)	81	16.7%
	University or more	56	11.6%
Marital status	Single	119	24.5%
	First marriage	337	69.5%
	Other	29	6.0%
Residency	Place of registration	336	69.3%
Monthly household expenditure, CNY	< 1000	58	12.0%
	1000–3000	255	52.6%
	3001–5000	132	27.2%
	> 5000	40	8.3%
Distance to TB clinic, km	0–9	170	35.1%
	10–29	203	41.9%
	30–39	45	9.3%
	40+	67	13.8%
Distance to local village/township doctor, km	0–1.9	247	50.9%
	2–2.9	109	22.5%
	3+	129	26.6%
Smear status	Positive	309	64.1%

## Discussion

This large, pragmatic trial has been designed to determine whether a new patient management model, based around the MERM, results in improved clinical outcomes for TB patients. The aim of the trial is to inform government policy in China, and elsewhere, and is thus fundamentally a study of effectiveness rather than efficacy. For this reason, we have elected to conduct a highly pragmatic trial.

**Fig. 3:**
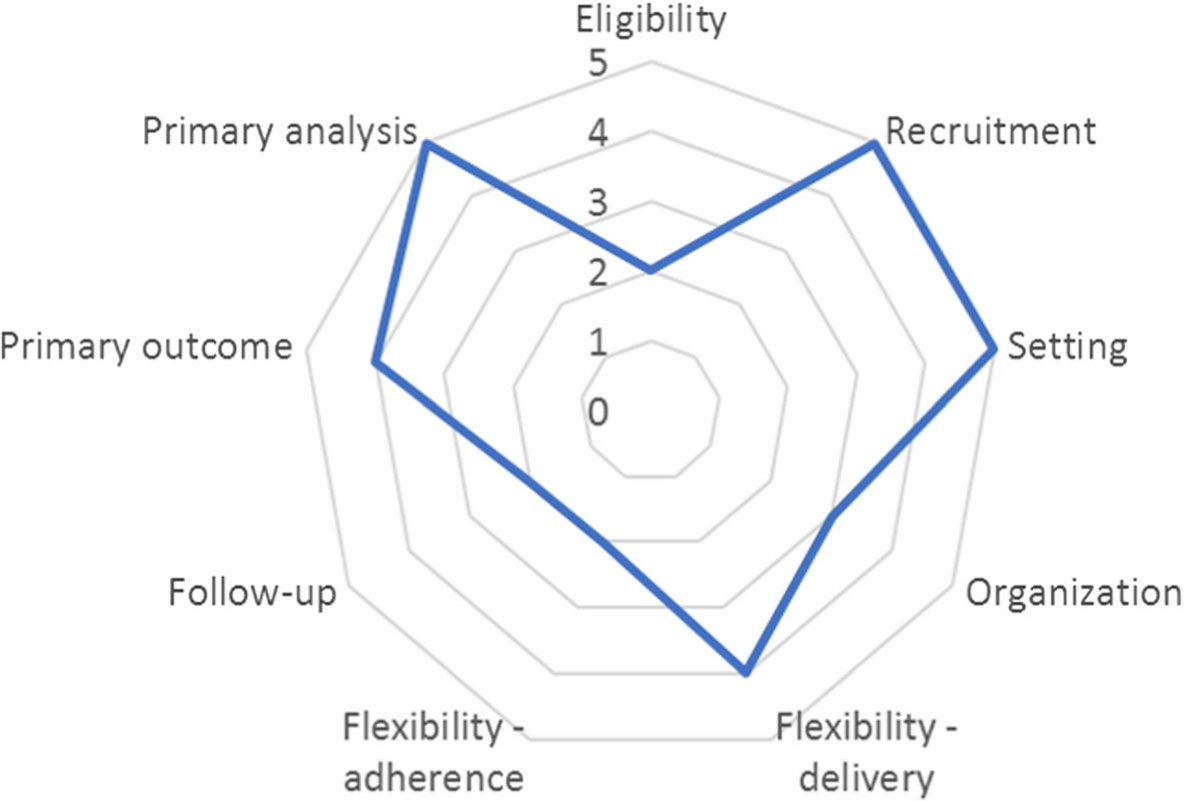
Domain scores using the PRECIS-2 tool to determine where the trial lies on the pragmatic/explanatory continuum (1 = highly explanatory to 5 = highly pragmatic)

We have scored our trial using the PRagmatic Explanatory Continuum Indicator Summary (PRECIS-2) tool, which describes where a trial lies on the pragmatic/explanatory continuum across nine domains [15]. Two authors (JL and SS) scored the trial on each of the nine domains and discrepancies were resolved by a third author (KF). The trial scored as ‘very pragmatic’ on three domains, namely ‘recruitment’, ‘setting’ and ‘primary analysis’, and as ‘rather explanatory’ on three domains, namely ‘eligibility’, ‘flexibility – adherence’ and ‘follow-up’ (Fig. 3). The intervention is applicable for almost all TB patients using fixed-dose combination tablets, and so most of the eligibility criteria result from the needs of the trial. The primary outcome is measured over 18 months; therefore, we want to ensure that participants are planning on staying in the area for the next 18 months. To ensure comparability between participants, we also require them to be on treatment for the same duration, namely 6 months. We want to ensure that all participants have drug-sensitive TB to ensure the best chance of impacting a clinical outcome. The intervention is designed to maximise adherence to the intervention; thus, we have scored the ‘flexibility – adherence’ domain as ‘rather explanatory’, as suggested in the guidance [15]. All treating doctors are trained on the study procedures, but we do not enforce any aspect of the protocol, in line with the desire to make the trial as pragmatic as possible. There are three reasons why we scored ‘follow-up’ as ‘rather explanatory’; firstly, our primary outcome requires a follow-up visit at 6 and 12 months after the end of treatment; second, we will contact participants by telephone at 3 and 9 months after the end of treatment to see if they have restarted TB treatment; and finally, in some provinces, TB treatment now requires clinic visits less than monthly, which we require in the control arm. The average score across the nine domains was 3.6, with 1 being ‘very explanatory’ and 5 being ‘very pragmatic’.

Since the publication of the systematic review by Nglazi et al. [7], two randomised controlled trials of an mHealth intervention for TB have reported their results. An individually randomised trial of a two-way SMS reminder system in Pakistan, using an intervention similar to our first trial, showed no impact on clinically recorded treatment success (83% in intervention arm vs. 83% in control arm) or self-reported medication adherence [16]. In contrast, a cluster randomised trial of a one-way SMS reminder system in China showed an improvement in treatment completion (96% in intervention arm vs. 87% in control arm), although the clustered design was not accounted for in the analysis [17].

In Table 3, we summarise six ongoing randomised trials, including our own, which are assessing eHealth interventions to improve TB treatment-related outcomes and treatment adherence. All trials were identified through a search of trial registries. In addition to our study, two are assessing VOT and three daily SMS reminders, total sample sizes vary from 260 to 3000, and inclusion criteria are generally very broad. Promisingly, all studies are measuring end-of-treatment outcome, rather than solely adherence outcomes, though not necessarily as the primary endpoint. However, many of the studies are measuring end-of-treatment outcomes using programmatic data. Treatment success combines cure and treatment completion, but often the programmatic data result in completion being the more common of the two. Arguably, this provides poor quality data on patient cure. Of note, ours is the only trial that we are aware of that is using a clinical endpoint that includes microbiological data from beyond the end of treatment, as is common in trials of new TB regimens [12–14].

**Table 3:** Summary of ongoing randomised controlled trials of eHealth interventions for TB

Study number^1^	Yr^2^	End date	Country	I/C^3^	Sample size	Study population	Intervention-eHealth for adherence	Control	Primary outcome	Other relevant outcomes
1	2017	2017	Kenya	I	1200	Any age; clinically diagnosed with TB by smear microscopy, culture or GeneXpert; has access to mobile phone	Daily request for self-verification of drug intake; Messages via ‘Keheala’ using text message-like interactions	Patients receive medication for 1–2 w; assigned a friend or family member supporter to verify the patient’s drug intake and return to the clinic with patient for refills	Unsuccessful treatment outcomes	–
2	2014	2017	Moldova	I	400	18+ y; at least 4 m of care remaining; not homeless, in prison, alcoholic/drug users, on injectables	VOT – daily observation of drug intake observed via internet video messages; VOT observers view and respond to video messages sent by patients	DOT – patient goes to polyclinic to be observed taking treatment every day	Adherence to medication	Adherence 80%; treatment success (measured at 4 months); side effects reported during treatment
3	2014	2015^4^	Armenia	C	380	18+ y; diagnosed with drug-sensitive TB and completed intensive phase	Daily SMS reminders to TB patients	DOT – observed taking treatment 6 days/w by healthcare provider	TB treatment success (cured/completed treatment) according to WHO definitions	TB treatment adherence by self-report
4	2013	2014^4^	Cameroon	I	260	18+ y; smear positive pulmonary TB, have a mobile phone and able to receive and open SMS	Daily SMS reminders to take TB drugs; content of messages changes every 2 weeks	Patients attend appointments for drug supplies weekly/monthly in intensive phase and monthly for continuation phase; SMS sent at start and at end of treatment	Treatment cure (smear-negative) at 6 m	Treatment adherence measured by VAS and appointments attended at 2, 5, and 6 m; treatment failure at 5 m; number of patients who develop resistance at 5 and 6 m
5	2014	2016	United Kingdom	I	400	16+ y; any TB patient from participating clinics who is eligible for DOT	VOT clips submitted using a dedicated smartphone with a pre-loaded app; VOT clips read by a study nurse/VOT observer daily during weekdays, weekend clips read on Mondays	DOT – by clinic staff, community-based (responsible professional: hostel worker/pharmacist) or by outreach worker; every day or weekdays and self-administered at weekend	Proportion of participants having more than 80% of scheduled VOT/DOT sessions successfully completed in the 2 m following randomisation	Proportion of doses observed over 2 and 6 m; culture conversion at 2 m; treatment outcome at 12 m acquisition of new resistance; and membership of a transmission cluster
6	2016	2019	China	C	3000	18+ y; Xpert positive (RIF sensitive), on fixed dose combination	Patients are provided with MERM box with reminding functions (audio and light) for (i) daily drug-intake and (ii) attendance of monthly follow-up appointments	Standard of care – self-administered, family- or healthcare worker-supported; MERM in silent mode	Composite unfavourable outcome: death, loss to follow-up, treatment failure, treatment between the end of treatment and 18 m after enrolment	End of treatment outcomes; adherence outcomes

^1^Additional details of each study: (1) Trial registration at NCT03135366; intervention also includes access to a supporter via a chat client, and information about TB. (2) Trial registration at NCT02331732. (3) Trial registration at NCT02082340; Trial protocol: Khachadourian et al. [18]. Intervention also includes the following: (i) education and counselling session for drug-sensitive TB patients and their family members (90 min); (ii) self-administered drug intake supervised by trained family member; (iii) daily phone calls to supporting family member; (iv) patients receive weekly SMS messages to attend the clinic weekly to receive their medication; Cluster randomised – cluster is defined as a TB outpatient centre; 52 clusters in total. (4) Trial registration at PACTR201307000583416; Trial protocol: Bediang [19]. (5) Trial registration at ISRCTN26184967. (6) Trial registration at ISRCTN35812455. Intervention also includes the doctor downloading the monthly drug intake recorded from MERM and assessing how many doses have been missed, with patient. Based on the missed doses, additional interventions are recommended to be implemented by the patient’s doctor such as additional visits from the township/village doctor; Cluster randomised – cluster is defined as a county/district; 24 clusters in total

^2^Year trial was registered

^3^I/C individually (I) or cluster randomised (C)

^4^As reported in the trial registration

*DOT* directly observed treatment, *m* month, *MERM* medication event reminder monitor, *RIF* rifampicin, *SMS* short message service, *TB* tuberculosis, *VOT* video-observed treatment, *VAS* visual analogue scale, *w* week, *y* years

An important consideration in trials of new treatment models for TB relates to the choice of control arm. The control arm differs across the six ongoing trials, which included three trials with DOT, one with a friend or family supporter, one with self-administered treatment and our trial, which uses standard-of-care as a mix of all three options. It could be argued that the control arm should be based on DOT, as recommended by the WHO. However, as our aim is to provide evidence for policy, we decided to make the pragmatic choice of using standard-of-care as the control arm. If a rigorously implemented control arm is based on DOT, then arguably such a trial should be conducted as a non-inferiority trial, as it would be difficult to improve on rigorously implemented DOT. In such a trial, the aim would likely be to determine whether the new treatment model is as good as (that is non-inferior to) DOT, but presumably with a lower burden on the health system. However, all of the published and ongoing trials are being conducted as superiority trials, including those directly comparing VOT to DOT.

Both this trial and our previous trial have randomised at the cluster level, rather than at individual patient level, as has one of the five other ongoing trials summarised and one other published trial [17] (Table 3). Randomising at the cluster level has the obvious disadvantages of increased sample size and increased possibility of allocation bias. However, we felt that these were outweighed by the reduction in logistical complexity and reduction in possible contamination between trial arms gained by randomising at the level of the health system that treats patients (in China this is at the county/district level).

Our trial will provide rigorous evidence of the impact of a health system intervention based around a medication monitor on clinical outcomes in China. Several other trials are investigating the potential of other eHealth interventions, making this an exciting time for the emerging evidence base on eHealth interventions for TB. We believe that our consideration of trial design issues presented in this paper is a useful addition to the literature on evaluation of eHealth interventions in this emerging field.

## Trial status

The protocol is version 3.0, dated September 21, 2016. The trial started recruitment to the run-in period on November 1, 2016, and to the main trial on January 16, 2017. Recruitment to the main trial is ongoing and is expected to be completed by December 31, 2018.

## Additional files

Additional file 1: Informed consent form and patient information sheet. (DOCX 33 kb)

Additional file 2: Completed SPIRIT checklist. (DOCX 59 kb)

## Abbreviations

aMR: Adjusted mean ratio
CI: Confidence interval
DOT: Directly observed treatment
MERM: Medication event reminder monitor
SMS: Short message service
TB: Tuberculosis
VOT: Video-observed treatment
WHO: World Health Organization
