# Supplementary material for: Evaluation of a medication monitor-based treatment strategy for drug-sensitive tuberculosis patients in China: study protocol for a cluster randomised controlled trial
Source: Trials. 2018 Jul 25;19:398. doi: 10.1186/s13063-018-2650-3 (PMC6060502; doi:10.1186/s13063-018-2650-3)
Supplement: Supplementary file 1 — Informed consent form and patient information sheet. (DOCX 33 kb) [file 13063_2018_2650_MOESM1_ESM.docx]

**Participant information sheet: electronic medication box arm – MAIN TRIAL**

**Cluster randomized trial of a medication monitor in the treatment management of patients with pulmonary tuberculosis**

**Trial run by:**

National Center for TB Control and Prevention, China Center for Disease Control and Prevention

*Protocol version 3.0, 21 September 2016*

We are doing a research study and we would like to invite you to take part. Research is the process to learn the answer to a question. This information sheet explains the study. You are free to decide if you want to take part or not. If you decide to take part, we will ask you to sign the consent form. By doing this, it means that you agree to take part in the study, and that you are aware of your right not to take part, or to stop taking part at any time. Your decision to take part or not will not affect your health care.

**Why are we doing this study?**

- Tuberculosis is curable under standard treatment management. However, due to irregular medication or interruption of the course, drug-resistant TB is developed among a number of patients. This makes their condition worse and results in the tuberculosis being difficult to cure. To make sure TB treatment adherence is good, the National Center for Tuberculosis Control and Prevention, China CDC, is conducting a community trial of a new method in tuberculosis treatment management.
- All TB patients in this district/county are invited to enrol in the study. A patient’s involvement in the study will last for 18 months from the start of treatment.
- Patients will receive an electronic medication box. Their treatment management is conducted with the use of electronic medication box. The electronic medication box will remind TB patients when to take their medication by using a sound alarm and green light. Patients will also be reminded when to return to the TB dispensary for monthly re-fills, using a yellow light, and for when to give spit specimens at 2, 5 and 6 months following start of TB treatment using pictograms.
- The electronic medication box also records when the box was opened. At the monthly visits to the TB dispensary if information from the electronic medication box suggests a TB patient is having problems taking their medication then other methods will be used to help the patient.
- Patients will also have a chest x-ray at 6 months from the start of treatment, and be asked to give spit specimens and have a chest x-ray at 12 and 18 months from the start of treatment (approximately 6 and 12 months after the end of treatment). This is to make sure that TB has not come back. We will telephone patients 9 and 15 months from the start of treatment (approximately 3 and 9 months after the end of treatment) to check your health and ask whether you have restarted TB treatment since we last spoke to you.
- TB patients in other districts/counties are receiving standard of care. We will then compare treatment adherence amongst patients from districts/counties receiving the electronic medication box to improve TB adherence with patients receiving standard of care.
- Around 3200 TB patients from 24 districts/counties in Zhejiang, Jiangxi and Jilin provinces will enrol in the study.
- The study is funded by the Gates Foundation. The funder is not the manufacture of the electronic medication box and will not make a profit from it.
- The overall study duration is from August 2016 to June 2019.

**If you take part in this study, what will happen?**

If you agree to take part in this study, with your permission:

- We will ask you some questions about yourself (such as your age, where you work, family income and your education), your TB diagnosis (distance to the dispensary) and measure you height and weight.
- As part of routine care you will be asked to return to the TB dispensary for monthly re-fills of your TB medication and to return to the TB dispensary at 2, 5 and 6 months following start of TB treatment to give spit (sputum) specimens.
- You will be given an electronic medication box to store your TB drugs. The electronic medication box records when you open the box. The electronic medication box also helps you to take your TB drugs on time by using a sound alarm and green light at the time you need to take your drugs. You will also be reminded when to return to the TB dispensary for monthly re-fills, using a yellow light, and when to give spit specimens at 2, 5 and 6 months following start of TB treatment using pictograms.
- We will ask you to bring the electronic medication box to the monthly visits to the TB dispensary. If at these monthly visits information from the electronic medication box suggests you are having problems taking your TB drugs then other methods will be used to help you. These other methods may include more health information or health clinic staff may visit you in your home or you may be asked to go to the local health clinic to take your TB drugs.
- At the end of the 6 months of TB treatment we will take a chest x-ray and ask you a few more questions about whether you liked using the electronic medication box and if someone (such as a family member or friend) reminded you when to take TB drugs or helped you with the electronic medication box.
- At 12 and 18 months from the start of TB treatment (this is approximately 6 and 12 months after the end of treatment) we will ask you to come to the TB dispensary to give a spit specimen and to have a chest x-ray. This is to check that TB has not come back. We will also ask you a few questions about your current health at these visits.
- Your TB drugs and chest x-rays are free of charge. You do not have to pay to take part in this study. We will not pay you to take part in the study. We will provide you with transport costs for coming to the clinic at 12 and 18 months from the start of TB treatment.
- We would like your permission to use the information collected on your TB record card and other medical record data related to your TB. This information will be linked to other data collected and will be identified by a study number, not by your name
- All TB patients in this district/county are invited to enrol in the study. A patient’s involvement in the study will last for 18 months from the start of treatment, but their involvement after the end of TB treatment will be limited to two short follow-up visits (12 and 18 months from the start of treatment) and two telephone calls (9 and 15 months from the start of treatment).
- This study will take about 30 minutes of your time today, and about 15 minutes for the follow-up visits at the end of treatment and 12 and 18 months from the start of treatment. We will have a brief telephone call with you at 9 and 15 months from the start of treatment.
- We would also like your permission to use the information we collect from this study for other research studies to help us understand TB better. We would only do this if the ethics committees who protect the interests of people taking part in our studies first approved the other research studies.

**What are the risks and benefits of taking part in this study?**

The benefits of taking part in this study are:

- Doctors caring for you will receive information early on how well you have taking your medication and if you are having problems then different methods will be started. This will be a benefit to you as it will help ensure that there is no delay in your cure
- if the study shows that this is an effective way to treat TB, this method of reminding TB patients to take their TB drugs may be used more widely to help people with TB.
- By testing your spit for TB at 12 and 18 months and taking a chest x-ray we will be able to see if your TB has come back and enable you to get the appropriate treatment faster.

There are no major risks in taking part in the study.

**What happens if I do not agree to take part in this study?**

You do not have to take part in this study: if you do not take part, this will not affect the medical care that you receive. You can decide to stop taking part in the study at any time, without giving a reason.

**How will the information collected during this study be kept confidential?** Information from the electronic pill box on when you took your TB drugs will be used by the doctors at the TB dispensary to see if you are having problems with taking your drugs.

All other information collected during the course of this study will be kept securely and confidentially. Your name and contact details will only be available to study staff, and will be stored electronically, completely separately from the other information that we collect.

The information you give us will be identified on forms and on computer files only by a study number, not your name. This information may be reviewed by the Ethics Committee and independent monitors, to check that the study procedures were done correctly and the information is correct. Your information will remain confidential. Reports about the study and results that may be published in scientific journals will not include any information which allows you to be identified.

**Are there reasons why the intervention might be stopped early?**

We will stop the intervention if:

- you move away from this district/county
- the study is cancelled
- there could be other reasons we don’t know about yet.

You can stop taking part in the study at any time. If you want to stop taking part, just tell one of the study team at any time.

**What if I have more questions I wish to ask about this study?**

If you have any questions about this study, please ask us now. If you have questions later you can ask study staff, or telephone *Dr Xiaoqiu LIU [010-58900515 or 13671298087].*

The committees giving ethical approval for this study are the Research Ethics Committees of the Chinese Center for Disease Control and Prevention, China and of the London School of Hygiene & Tropical Medicine, UK.

We will give you a copy of this sheet which explains the study to take away with you.

**INFORMED CONSENT FOR CLUSTER RANDOMIZED TRIAL OF A MEDICATION MONITOR IN THE TREATMENT MANAGEMENT OF PATIENTS WITH PULMONARY TUBERCULOSIS**

**(MEDICATION MONITOR ARM – MAIN TRIAL)**

Trial run by

National Center for TB Control and Prevention, China Center for Disease Control and Prevention

Protocol version 3.0, 21 September 2016

*Participant unique identifier:*

1. I, ______ (insert name) have read the patient information sheet and have had the opportunity to consider the information, ask questions and have had these answered fully by (*insert name here*) _________________________
2. I understand my participation is voluntary and I am free to withdraw at any time, without giving any reason, without my medical care or legal rights being affected.
3. I agree to the researchers accessing my TB medical records
4. I agree to take part in the study and am willing to accept the use of electronic medicine box in the treatment management.
5. I agree to the researchers testing my spit for tuberculosis at 12 and 18 months after I start treatment
6. I agree to having a chest x-ray 6, 12 and 18 months after I start treatment

Patient’s Signature: ________________ Date: ______________

I have accurately informed the subjects of this document to the patients/recipients. He/she has accurately read informed consent and has the opportunity to raise question. I, hereby, guarantee that he/she sign this informed consent based on the voluntary principles.

Doctor’s (Name of Person taking consent) Signature: ___________ Date: ______________

**Participant information sheet: control arm – MAIN TRIAL**

**Cluster randomized trial of a medication monitor in the treatment management of patients with pulmonary tuberculosis**

**Trial run by:**

National Center for TB Control and Prevention, China Center for Disease Control and Prevention.

*Protocol version 3.0, 21 September 2016*

We are doing a research study and we would like to invite you to take part. Research is the process to learn the answer to a question. This information sheet explains the study. You are free to decide if you want to take part or not. If you decide to take part, we will ask you to sign the consent form. By doing this, it means that you agree to take part in the study, and that you are aware of your right not to take part, or to stop taking part at any time. Your decision to take part or not will not affect your health care.

**Why are we doing this study?**

- Tuberculosis is curable under standard treatment management. However, due to irregular medication or interruption of the course, drug-resistant TB is developed among a number of patients. This makes their condition worse and results in the tuberculosis being difficult to cure. To make sure TB treatment adherence is good, the National Center for Tuberculosis Control and Prevention, China CDC, is conducting a community trial of new methods in tuberculosis treatment management.
- All TB patients in this district/county are invited to enrol in the study. A patient’s involvement in this study will last for 18 months from the start of treatment.
- Patients will receive an electronic medication box which records when the box is opened. Their treatment management is conducted using standard of care in terms of national policy.
- Patients will also have a chest x-ray at 6 months from the start of treatment, and to give spit specimens and have a chest x-ray at 12 and 18 months from the start of TB treatment (approximately 6 and 12 months after the end of treatment). This is to make sure that TB has not come back. We will telephone patients 9 and 15 months from the start of treatment (approximately 3 and 9 months after the end of treatment) to check your health and ask whether you have restarted TB treatment since we last spoke to you.

- TB patients in other districts/counties are receiving another method to improve TB adherence. We will then compare treatment adherence amongst patients from districts/counties receiving the other method to improve TB adherence with patients receiving standard of care.
- Around 3200 TB patients from 24 districts/counties in Zhejiang, Jiangxi and Jilin provinces will enrol in the study.
- The study is funded by the Gates Foundation. The funder is not the manufacture of the electronic medication box and will not make a profit from it.
- The overall study duration is from August 2016 to June 2019.

**If you take part in this study, what will happen?**

If you agree to take part in this study, with your permission:

- We will ask you some questions about yourself (such as your age, where you work, family income and your education), your TB diagnosis (distance to the dispensary) and measure you height and weight.
- As part of routine care you will be asked to return to the TB dispensary for monthly re-fills of your TB medication and to return to the TB dispensary at 2, 5 and 6 months following start of TB treatment to give spit (sputum) specimens.
- You will be given an electronic medication box to store your TB drugs. The electronic medication box records when you open the box.
- We will ask you to bring the electronic medication box to the TB dispensary at the end of treatment or before if you have stopped treatment early.
- At the end of the 6 months of TB treatment we will take a chest x-ray and ask you a few more questions about whether you liked using the electronic medication box and if someone (such as a family member or friend) reminded you when to take TB drugs.
- At 12 and 18 months from the start of TB treatment (this is approximately 6 and 12 months after the end of treatment) we will ask you to come to the TB dispensary to give a spit specimen and to have a chest x-ray. This is to check that TB has not come back. We will also ask you a few questions about your current health at these visits.
- Your TB drugs and chest x-rays are free of charge. You do not have to pay to take part in this study. We will not pay you to take part in the study. We will provide you with transport costs for coming to the clinic at 12 and 18 months from the start of TB treatment.
- We would like your permission to use the information collected on your TB record card and other medical record data related to your TB. This information will be linked to other data collected and will be identified by a study number, not by your name
- All TB patients in this district/county are invited to enrol in the study. A patient’s involvement in the study will last for 18 months from the start of treatment, but their involvement after the end of TB treatment will be limited to two short follow-up visits (12 and 18 months from the start of treatment) and two telephone calls (9 and 15 months from the start of treatment).
- This study will take about 20 minutes of your time today, and about 15 minutes for the follow-up visits at the end of treatment and 12 and 18 months from the start of TB treatment. We will have a brief telephone call with you at 9 and 15 months from the start of treatment.
- We would also like your permission to use the information we collect from this study for other research studies to help us understand TB better. We would only do this if the ethics committees who protect the interests of people taking part in our studies first approved the other research studies.

**What are the risks and benefits of taking part in this study?**

The benefits of taking part in this study are:

- If the study shows that this is an effective way to treat TB, this method of reminding TB patients to take their TB drugs may be used more widely to help people with TB.
- By testing your spit for TB at 12 and 18 months and taking a chest x-ray we will be able to see if your TB has come back and enable you to get the appropriate treatment faster.

There are no major risks in taking part in the study.

**What happens if I do not agree to take part in this study?**

You do not have to take part in this study: if you do not take part, this will not affect the medical care that you receive. You can decide to stop taking part in the study at any time, without giving a reason.

**How will the information collected during this study be kept confidential?**

All information collected during the course of this study will be kept securely and confidentially. Your name and contact details will only be available to study staff, and will be stored electronically, completely separately from the other information that we collect.

The information you give us will be identified on forms and on computer files only by a study number, not your name. This information may be reviewed by the Ethics Committee and independent monitors, to check that the study procedures were done correctly and the information is correct. Your information will remain confidential. Reports about the study and results that may be published in scientific journals will not include any information which allows you to be identified.

**Are there reasons why the intervention might be stopped early?**

We will stop the intervention if:

- you move away from this district/county
- the study is cancelled
- there could be other reasons we don’t know about yet.

You can stop taking part in the study at any time. If you want to stop taking part, just tell one of the study team at any time.

**What if I have more questions I wish to ask about this study?**

If you have any questions about this study, please ask us now. If you have questions later you can ask study staff, or telephone *Dr Xiaoqiu LIU [010-58900515 or 13671298087].*

The committees giving ethical approval for this study are the Research Ethics Committees of the Chinese Center for Disease Control and Prevention, China and of the London School of Hygiene & Tropical Medicine, UK.

We will give you a copy of this sheet which explains the study to take away with you.

**INFORMED CONSENT FOR CLUSTER RANDOMIZED TRIAL OF A MEDICATION MONITOR IN THE TREATMENT MANAGEMENT OF PATIENTS WITH PULMONARY TUBERCULOSIS**

**(CONTROL ARM– MAIN TRIAL)**

Trial run by

National Center for TB Control and Prevention, China Center for Disease Control and Prevention.

Protocol version 3.0, 21 September 2016

*Participant unique identifier:*

1. I, ______ (insert name) have read the patient information sheet and have had the opportunity to consider the information, ask questions and have had these answered fully by (*insert name here*) _________________________
2. I understand my participation is voluntary and I am free to withdraw at any time, without giving any reason, without my medical care or legal rights being affected.
3. I agree to the researchers accessing my TB medical records
4. I agree to take part in the study and accept the use of electronic medicine box
5. I agree to the researchers testing my spit for tuberculosis at 12 and 18 months after I start treatment
6. I agree to having a chest x-ray 6, 12 and 18 months after I start treatment

Patient’s Signature: ________________ Date: ______________

I have accurately informed the subjects of this document to the patients/recipients. He/she has accurately read informed consent and has the opportunity to raise question. I, hereby, guarantee that he/she sign this informed consent based on the voluntary principles.

Doctor’s (Name of Person taking consent) Signature: ___________ Date: ______________
